# Taxonomy and nomenclature of the polymorphic European high mountain species *Androsace vitaliana* (L.) Lapeyr. (Primulaceae)

**DOI:** 10.3897/phytokeys.75.10731

**Published:** 2016-12-06

**Authors:** Christopher J. Dixon, Walter Gutermann, Peter Schönswetter, Gerald M. Schneeweiss

**Affiliations:** 1 Department of Botany and Biodiversity Research, University of Vienna, Rennweg 14, A–1030 Wien, Austria; 2 Institute of Botany, University of Innsbruck, Sternwartestrasse 15, A-6020 Innsbruck, Austria

**Keywords:** Androsace vitaliana, Bayesian species delimitation, European mountain ranges, nomenclature, typification, Vitaliana primuliflora

## Abstract

*Androsace vitaliana* (syn. *Vitaliana primuliflora*; Primulaceae) has been subject to several taxonomic treatments, whose conclusions ranged from a single species with numerous infraspecific taxa to several species usually without infraspecific taxa. Here, following molecular investigation, several taxonomic changes are made. A single species with the following infraspecific taxa is recognized: subsp. *vitaliana* (Pyrenees), subsp. cinerea (south-western Alps), subsp. lepontina (Pennine Alps), subsp. sesleri (south-eastern Alps), subsp. praetutiana (Apennines) and subsp. assoana (Iberian Peninsula excluding the Pyrenees), the last of which is divided into the four allopatrically distributed varieties *assoana*, *centriberica*, *flosjugorum* and *nevadensis*. Contrary to some previous assertions, all taxa are allopatric and, especially for subspp. *vitaliana*, *cinerea* and *lepontina*, where clear diagnostic characters are lacking, they can best be determined by their geographic origin.

## Introduction

The infraspecific taxonomy of *Androsace vitaliana* (L.) Lapeyr. has been studied by several authors (Lüdi 1927, Chiarugi 1930, Schwarz 1963, Kress 1967, 1997a, 1999), who have reached different conclusions, based on morphological differences, geographical distributions and a certain amount of intuition. Recent genetic studies of *Androsace vitaliana* by Vargas (2003) and Dixon et al. (2009) offered new and unexpected insights into the species’ phylogeny. Based on the results of these works and new analyses presented here, we now present a new taxonomy of *Androsace vitaliana*.

## Taxonomic history

The first infraspecific taxa to be distinguished in *Androsace vitaliana* (under the name *Gregoria Vitaliana*—for the nomenclatural evaluation of the names that have been used and for full nomenclatural authorities, see “Nomenclature”) were the three varieties named by Sündermann (1916), although he ascribed two of them to Robert Buser. He defined var. praetutiana from the central Apennines by its relatively short leaves with rounded tips and by the density of the indumentum, and var. cinerea from the south-western Alps by the size and colour of the flowers, the shape of the leaves, and the ash-grey tomentum. Sündermann characterised the south-east Alpine var. sesleri by its slightly broader and greener leaves, but did not consider it to be very different from the “typical” form supposed to occur in the western Alps. Lüdi (1927) followed Sündermann’s classification, but reduced the varieties to the rank of forma.

Chiarugi (1930) divided the species (as *Vitaliana primuliflora*) into three varieties: plants from the Sierra Nevada (var. nevadensis) form dense cushions and have persistent, distinctly keeled leaves tightly arranged on columnar stems; plants from the Apennines (var. praetutiana) form loose cushions or mats and have deciduous, broader leaves with rounded leaf tips; plants from the Pyrenees and the Alps (var. alpina) also form loose cushions or mats, but their leaves are narrower and have acute tips. Due to a lack of material, Chiarugi did not assign plants from the Sierra de Javalambre (eastern Spain) to any of his infraspecific taxa. Within the Pyrenean–Alpine taxon, he distinguished the eastern f. orientalis (Pennine Alps to southeastern Alps), with hairs either only on the leaf margins or on both the leaf margins and the median nerve on the lower leaf surface, from the western f. occidentalis (Pyrenees and south-western Alps), with hairs dispersed over at least the lower but commonly both leaf surfaces. Chiarugi further divided these formae into two subformae each, but the morphological differences were only reported to coincide with a geographical separation in f. orientalis, where the south-eastern Alpine taxon (subf. tridentina) had the least hairy leaves, while the taxon in the Pennine Alps (subf. lepontina) had hairier leaves. In contrast, for the two taxa within f. occidentalis, subf. genuina with longer corolla tubes and subf. cinerea with shorter corolla tubes, Chiarugi himself was unable to determine the exact distributional limits, and he only noted that both were present in both the eastern Pyrenees and the southwestern Alps. Later authors (e.g., Schwarz 1963, Kress 1997a, b) have been more specific about the distributions in the Pyrenees, limiting one taxon to the central parts of the range and the other to the eastern parts.

In his taxonomic revision, Schwarz (1963) divided *Androsace vitaliana* (as the genus *Vitaliana*) into five species. While the distinctness of the Apennine taxon (*Vitaliana obtusifolia*) and the taxon from the Sierra Nevada (*Vitaliana congesta*) remained undisputed, populations from the Sierra de Javalambre were separated as a new species, *Vitaliana intermedia*, based on characters such as keeled leaves with obtuse tips and the shape of the calyx lobes, thus extending Chiarugi’s observation of keeled leaves to cover the Sierra de Javalambre as well as the Sierra Nevada. Schwarz distinguished those plants that have leaves that are hairy on both sides (as *Vitaliana chionotricha*) from those with leaves that are glabrous on at least the upper surface (as *Vitaliana primuliflora*), dividing the latter into two subspecies, one with hairy lower leaf surfaces (subsp. canescens) and the other with both leaf surfaces glabrous (subsp. primuliflora). Although *Vitaliana chionotricha* and Vitaliana primuliflora subsp. canescens were considered to occur in both the Pyrenees and the western Alps (Schwarz’ subsp. primuliflora is the taxon from the south-eastern Alps), Schwarz suggested that these are not sympatric, with *Vitaliana chionotricha* confined to the eastern Pyrenees and the south-western Alps, and Vitaliana primuliflora subsp. canescens occurring in the central Pyrenees and the Pennine and Lepontine Alps (see Fig. 6 in Schwarz 1963: p. 28). The Alpine distribution area of subsp. canescens is thus congruent with that of Chiarugi’s f. orientalis subf. lepontina (see above). However, although Schwarz’ map shows a clear geographic division between the two taxa, Kress (1967) asserts that the distributions are not as clearly separated as shown on Schwarz’ map.

Kress (1967), as well as taking a different view about the type material, which affects the use of the epithets *vitaliana* and *sesleri* (see “Nomenclature”), rejected the division of *Vitaliana* into five species. Instead, he considered the taxon to be a single species and returned it to the genus *Androsace*. Since this treatment was done for Hegi’s “Illustrierte Flora von Mitteleuropa”, Kress only commented on the Alpine taxa, listing them as subsp. cinerea (eastern Pyrenees and western Alps), subsp. sesleri (south-eastern Alps) and subsp. *vitaliana* (Pyrenees, western Alps). While providing a diagnostic key (based on Schwarz 1963), Kress commented that the values for the number of palisade layers in the upper leaf surface and the size of the flowers, characters used by previous authors (Sündermann 1916, Chiarugi 1930, Schwarz 1963), are not always correct. In a later publication, Kress (1997a) also stated that the shape of the leaves is highly dependent on the environmental conditions under which the plant grows, with drier environments giving rise to longer, proportionally narrower leaves, making this another unreliable character. Kress (1967) followed Schwarz’ view of the indumentum, albeit with some reservation about the uniformity of the character within and between populations. In contrast, Heß et al. (1970) dismissed the indumentum characters entirely, having observed that glabrous individuals could be found even in the southwestern Alps, where, according to the traditional viewpoint, plants should have hairs on at least the lower leaf surface.

Laínz (1964) combined specimens from the Cordillera Cantábrica and the Sierra de Javalambre as subsp. assoana. Since then, the major changes have been in the recognition of an increasing number of taxa on the Iberian Peninsula, and at increasing rank. Kress (1997a) described a new subspecies for plants from the Cordillera Cantábrica (subsp. flosjugorum, which forms loose cushions or mats and has a fraction of leaves that are distally hairy on the upper surface) as well as a variety in the Sistema Central (var. centriberica, which forms dense cushions with spreading dead leaves and juvenile leaves that have obtuse tips), which Luceño (1998) raised to the subspecific rank as subsp. aurelii. Kress (1999) raised the rank further, creating the species *Androsace centriberica*, with allopatric subspecies in the Sistema Central (subsp. centriberica), Sierra de Javalambre (subsp. assoana) and Montes de León (subsp. maragatorum, which differs from the previous two subspecies by more strongly hairy upper leaf surfaces and acute leaf tips), but excluding the Cordillera Cantábrica. At the same time, he also resurrected Schwarz’ species from the Sierra Nevada as *Androsace congesta*. This last work by Kress contradicts earlier works by the same author, introducing new taxa based on characters that he had previously deemed unreliable.

### Previous molecular findings

In the first molecular investigation of the species, using sequences from the nuclear ribosomal ITS region, Vargas (2003) was not able to resolve all the relationships within *Androsace vitaliana*, but did detect three distinct lineages: one in the Apennines, one in the Alps, and one on the Iberian Peninsula. Dixon et al. (2009), using additionally plastid sequences and AFLPs, identified six major groups, corresponding closely with the geographical distribution of the species. All Spanish accessions from outside the Pyrenees formed a single group, with only weak differentiation between the different mountain ranges. Kress’ (1999) opinion that these populations belong to three different species (*Androsace congesta*, *Androsace centriberica*, and *Androsace vitaliana*) found no support. No material from the Montes de León was included in the study of Dixon et al. (2009), but in Vargas’ (2003) study, the Cordillera Cantábrica and Montes de León grouped together in agreement with their geographic proximity. Populations from the Pyrenees constituted a single group (ribotype sharing with Spanish populations outside the Pyrenees was interpreted as the result of ITS convergence after gene flow into the Pyrenees: Dixon et al. 2009). In the Alps, three groups were identified: in addition to populations from the southwestern Alps (subsp. cinerea) and from the southeastern Alps (subsp. sesleri) populations from the Pennine Alps were found to be as distinct as the previous two or as subsp. praetutiana from the Apennines, supporting Chiarugi’s (1930) assessment that the populations from the Pennine Alps formed a distinct taxon. This disagrees with most recent treatments, in which plants from the Pennine Alps have been grouped with those from the central Pyrenees.

### Bayesian species delimitation

To further test the distinctness of the six major groups identified by Dixon et al. (2009), their AFLP data set was re-analysed via Bayesian multispecies coalescent species delimitation using genome-wide biallelic markers (Leaché et al. 2014). The applicability of this method may be compromised in case of substantial gene flow between diverging entities (populations, subspecies, species); high bootstrap support obtained from a Neighbour-Joining analysis for all major lineages except the one from the southwestern Alps (Fig. 3 in Dixon et al. 2009: p. 585), however, suggests that the evolution of this group can be reasonably well described by a tree. Applying this method allows competing species delimitation concepts to be compared using Bayes Factors (Kass and Raftery 1995). To decrease the computational burden, the original AFLP data set was pruned to include only two randomly chosen individuals per population, retaining 60 of the 143 individuals in the data set of Dixon et al. (2009). Analyses were conducted using the SNAPP 1.2.5 package (implementing the method of Bryant et al. 2012) in BEAST 2.1–2.3 (Bouckaert et al. 2014). Priors for forward and backward mutation rates, *u* and *v*, were fixed on 1.017056 and 0.983506, calculated from the state frequencies and applying the constraint that the mutation rate is 1. Priors for other parameters (λ for the Yule prior on species trees, α and β for the gamma distribution priors on the θ values of the ancestral population sizes) were fixed at their default values. Bayes Factors were calculated from marginal likelihoods obtained via path sampling (Baele et al. 2012) with 60 steps (each with 10^6^ MCMC steps and 50,000 pre-burnin steps). Tested species delimitation scenarios were: (i) all six major groups constitute distinct lineages; (ii) groups from the southwestern and Pennine Alps constitute a single lineage, while the remaining four are distinct; (iii) the three groups from the Alps constitute a single lineage, while the remaining three are distinct; (iv) the three groups from the Alps plus the Apennines constitute a single lineage, while the remaining two are distinct. Although the estimated marginal likelihoods need to be viewed with some caution (up to seven consecutive steps between step 20 and 30 of the path sampling had effective sampling size [ESS] values below 100), large positive Bayes Factors (i.e., twice the difference in marginal likelihoods between model 1 and model 2) provided strong support for the first scenario, i.e., recognition of all six major groups identified by Dixon et al. (2009) as distinct lineages (Table 1).

**Table 1.** Results for Bayesian lineage delimitation in *Androscae vitaliana*.

Model	Marginal likelihood	Bayes Factor
(i) Six lineages (Iberian Peninsula, Pyrenees, southwestern Alps, Pennine Alps, southeastern Alps, Apennines)	-2580.65	−
(ii) Five lineages (Iberian Peninsula, Pyrenees, southwestern plus Pennine Alps, southeastern Alps, Apennines)	-2745.68	330.05
(iii) Four lineages (Iberian Peninsula, Pyrenees, Alps, Apennines)	-2761.82	362.33
(iv) Three lineages (Iberian Peninsula, Pyrenees, Alps plus Apennines)	-2835.78	510.27

### Classification

Previous taxonomic treatments range from a very elaborate infraspecific classification down to the level of subformae (Chiarugi 1930) to ones using a very narrow species concept, which distinguish up to five different species (Schwarz 1963, Kress 1999). The prevailing view lies between these two extremes and treats *Androsace vitaliana* as a single species with several subspecies (Kress 1967, 1997b, Ferguson 1972, Pignatti 1982), a scheme we follow here for several reasons. Firstly, there is evidence for (ancient) gene flow between the different mountain ranges (Dixon et al. 2009); secondly, there is a lack of reliable diagnostic features that could be used for species discrimination, especially between taxa from the Pyrenees and the western Alps. Finally, this is the choice of rank that requires the fewest changes to the established names, thus helping to ensure nomenclatural stability.

Three subspecies possess distinctive morphological features that allow them to be distinguished, at least to some extent, from other taxa: subsp. praetutiana (Apennines) has rounded leaf tips (also found in some populations of subsp. assoana, but these form dense cushions, and rarely in subsp. *vitaliana*: Kress 1997a); subsp. assoana (Iberian Peninsula outside the Pyrenees) has keeled leaves (only weakly so in populations from the Cordillera Cantábrica), which tend not to fall off after dying away and give rise to thick columnar stems (in contrast to the slender stems seen in other taxa, but also in the populations of subsp. assoana from the Cordillera Cantábrica: Kress 1997a); subsp. sesleri (south-eastern Alps) has the least hairy leaves that are either glabrous or only sparsely hairy along the leaf margin (Schwarz 1963, Kress 1967). The remaining three subspecies have chiefly been defined in terms of their indumentum, but despite the presence of some morphological tendencies (subsp. cinerea is generally the most densely hairy taxon with the longest leaves, for instance), these characters are variable both within and between populations, and do not correlate with genetic relatedness. The easiest way to distinguish these subspecies remains, therefore, knowledge of their geographic origins: subsp. *vitaliana* occurs in the Pyrenees, subsp. cinerea in the south-western Alps, and subsp. lepontina in the Pennine Alps. We acknowledge that it is a disadvantage that these taxa cannot be confidently diagnosed from their morphology, but we consider it necessary to reflect our current understanding of their phylogenetic relationships and distinctness as far as possible in the taxonomy. The taxa from the Pyrenees and the western Alps are genetically as distinct as the other taxa that are better defined morphologically (Dixon et al. 2009). Although only the geographic location can currently be used to identify these taxa accurately, it is possible that suitable diagnostic characters exist, but have as yet been overlooked.

This problem also applies to the varieties within subsp. assoana, which are difficult to distinguish from herbarium material without knowledge of the geographic origin as acknowledged by Kress in his latest contribution (Kress 1999), ironically the very study where he uses the highest taxonomic ranks. However, given that diagnostic tendencies have been recognised by previous authors (Kress 1997a,b, 1999), we have chosen to preserve these varieties, in order to retain as much as possible of the existing nomenclature while also reflecting the genetic relationships discovered by Vargas (2003) and Dixon et al. (2009). Specifically, var. flosjugorum is distinguished by growing in loose cushions or mats in contrast to the other three varieties that form dense cushions; var. centriberica has slightly shorter and broader leaves than the other varieties, with a tendency to be rounded at the tip, while the leaves of var. assoana are narrower and have acute tips; var. nevadensis reportedly forms smaller cushions than the other varieties of subsp. assoana and has imbricate leaves that are only occasionally rounded.

### Nomenclature

*Androsace vitaliana* (L.) Lapeyr., Hist. Pl. Pyrénées: 94. 1813 ≡ *Primula vitaliana* L., Sp. Pl.: 143. 1753 [basionym] ≡ *Aretia vitaliana* (L.) L., Syst. Veg. ed. 13: 162. 1774 ≡ *Androsace lutea* Lam., Fl. franç. 2: 258. 1778, nom. superfl. ≡ *Primula sedifolia* Salisb., Parad. Lond. 2: t. 107. 1807, nom. superfl. ≡ *Androsace rugosa* Clairv., Man. herbor. Suisse: 57. 1811, nom. superfl. ≡ *Gregoria vitaliana* (L.) Duby, Bot. Gall. ed. 2, 1: 383. 1828 ≡ *Vitaliana primuliflora* Bertol., Fl. Ital. 2: 368. 1835 ≡ *Macrotybus luteus* Dulac, Fl. Hautes-Pyrénées: 425. 1867, nom. superfl. ≡ *Vitaliana gregorii* Saccardo, Sto. lett. fl. venet.: 46. 1869, nom. superfl. ≡ *Gregoria lutea* St.-Lager in Ann. Soc. Bot. Lyon, 7: 144. 1880, nom. superfl. ≡ *Douglasia vitaliana* (L.) Pax in Engler & Prantl, Nat. Pflanzenfam. 4(1): 109. 1890. — Lectotype [designated by Kress (1967: 2248d)]: Spain, Pyrenees, “*Sanicula alpina pumila tenuifolia lutea*, non descripta. In Pyrenaeis Hispanicis” in herb. Burser XIII.154 (UPS).

The Linnaean basionym *Primula vitaliana* has been repeatedly typified (Kress 1967, Ferguson 1969). Schwarz (1963) suggested as putative type material a specimen in the Linnaean herbarium in London (not explicitly designated by Schwarz, but identified as LINN 198-15 by Ferguson). However, this is a specimen that represents *Androsace alpina*, not *Androsace vitaliana*. Additionally, this accession has been acquired later than 1753, which is also the case for another specimen (LINN 196-4, preserved as *Aretia vitaliana*) of true *Androsace vitaliana* (Kress 1970). Kress (1967) identified a specimen held in Burser’s Hortus Siccus from the Spanish Pyrenees for the type specimen. As this specimen lacks flowers, Ferguson (1969) considered Kress’ selection unsatisfactory and instead favoured as lectotype a plate by Sesler, which was based on a specimen from the south-eastern Alps and shows flowers, descriptions of which are included in the protologue. Kress (1970) argues that Sesler’s drawing is chimerical, because it includes parts of *Silene acaulis* (Caryophyllaceae), and continues to favour the specimen held in Burser’s Hortus Siccus (Kress 1970, 1993). We follow Kress’ choice of type, accepting his argument that the current lack of flowers does not necessarily preclude the specimen from having once possessed some and having been used at the time of description. We also concur that the possibility of having at least part of the type material belonging to a different species or even family should be avoided.

One synonym of *Androsace vitaliana* given by both Chiarugi (1930) and Schwarz (1963) appears to result from a misunderstanding. Chiarugi cites “*Primula lutea* Vill., Cat. du Gard. de Strasbur., 121 (1807).” as a synonym of *Androsace vitaliana*, referring to Villars’ Catalogue méthodique des plantes du Jardin de l’École de Médecine de Strasbourg, where a footnote concerning “*Primula vitaliana*, *Fl. fr. III*, 450” appears at the end of the treatment of the genus *Primula*, adjacent to the name “*Primula lutea*, Vill.”, which (as is apparent from its typographic position) is cited here as synonym of *Primula auricula*. The footnote refers to the critical placement of *Primula vitaliana* between *Primula*, *Aretia*, and *Androsace*. Thus, it applies simply to the whole genus and cannot be seen as an indication of synonymy between *Primula lutea* and *Primula vitaliana*. Schwarz (1963) went further and erroneously interpreted the name *Primula lutea* as a recombination of Lamarck’s *Androsace lutea*, adding Lamarck to the citation as the author of the basionym. The name *Androsace lutea* is itself illegitimate, since Lamarck ought to have adopted the basionymic epithet *vitaliana*, and all recombinations of *Androsace lutea* are therefore new (and likewise illegitimate) names.

Androsace vitaliana subsp. vitaliana

-Vitaliana primuliflora var. alpina f. occidentalis subf. genuina Chiarugi in Nuov. Giorn. Bot. Ital. 37: 337. 1930, nom. inval. (ICBN 24.3), p. p.

-Vitaliana primuliflora subsp. canescens O. Schwarz in Feddes Repert. 67: 24. 1963, nom. inval. (without type indication), p. p. (Pyrenean plants only)

-*Vitaliana chionotricha* O. Schwarz in Feddes Repert. 67: 24. 1963, p. p. (Pyrenean plants only; type excluded)

Distribution. Pyrenees.

Following the findings of Dixon et al. (2009), and contrary to previous hypotheses, only one subspecies of *Androsace vitaliana* occurs in the Pyrenees. Because the type specimen of the species name is from the Pyrenees, the Pyrenean taxon must bear the autonym.

Androsace vitaliana subsp. cinerea (Sündermann) Kress in Hegi, Ill. Fl. Mitt.-Eur. 5 (3): 2248d. 1967 ≡ Gregoria vitaliana var. cinerea Sündermann in Allg. Bot. Z. 22: 59. 1916 [basionym] ≡ Gregoria vitaliana subsp. cinerea (Sündermann) Bornm. in Mitt. Thür. Bot. Ver. 37: 6, 1927 ≡ Gregoria vitaliana f. cinerea (Sündermann) Lüdi in Hegi, Ill. Fl. Mitt.-Eur. 5 (3): 1789, 1927 ≡ Vitaliana primuliflora subf. cinerea (Sündermann) Chiarugi in Nuov. Giorn. Bot. Ital. 37: 337, 1930 ≡ Vitaliana primuliflora subsp. cinerea (Sündermann) I. K. Ferguson in Taxon 18: 303, 1969 ≡ Vitaliana primuliflora var. cinerea (Sündermann) Vigo in Acta Bot. Barcinonensia 35: 435, 1983 — Type: none designated (described from Mercantour, south-western Alps)

= *Vitaliana chionotricha* O. Schwarz in Feddes Repert. 67: 24, 1963 — Type: In colle “Galibier” montium Delphinentium. Leg. Ozanon. 1858. (holotype: JE; isotypes: W, M).

-Gregoria vitaliana subsp. gaudini Sündermann ex Bornm. in Mitt. Thüring. Bot. Vereins 37: 6. 1927, nom. nud.

-Vitaliana primuliflora var. alpina f. occidentalis subf. genuina Chiarugi in Nuov. Giorn. Bot. Ital. 37: 337, 1930, nom. inval. p. p. (Alpine plants only)

Distribution. Alps south and west of Mont Blanc.

Sündermann (1916) did not designate a type specimen for his var. cinerea, but did give a location: “von den hohen Bergen zwischen Pietraporzio und St. Etienne in den Seealpen” (“high mountains between Pietraporzio and St. Etienne in the Maritime Alps”). Saint-Étienne-de-Tinée (Alpes-Maritimes, France) and Pietraporzio (Cuneo, Italy) lie on either side of the northern part of the Mercantour mountain range. We refrain from selecting a neotype until a collection from the immediate type region (between Col du Fer and Pas de Corborant) is available, preferably investigated by molecular means.

Since no material from the Savoyan or Graian Alps has been included by Dixon et al. (2009), it is difficult to know to which subspecies these populations would belong. Pignatti (1982) had extended the area of his “subsp. canescens” (which largely corresponds to our subsp. lepontina) to Valle Locana (thus including the Gran Paradiso massif), whereas Chiarugi considered this population to be part of his forma occidentalis (i.e., our subsp. cinerea). This area is contiguous with the south-west Alpine distribution of *Androsace vitaliana*, and not with the area in the Pennine Alps (Niklfeld et al., unpubl. data). We therefore assume, in the absence of any genetic data, that these plants belong to subsp. cinerea.

Androsace vitaliana subsp. lepontina (Chiarugi) Dixon, Gutermann & Schneew. comb. nov. ≡ Vitaliana primuliflora var. alpina f. orientalis subf. lepontina Chiarugi in Nuov. Giorn. Bot. Ital. 37: 337, 1930 — Lectotype (here designated): Switzerland, Valais, “Seehorn a. d. Südseite d. Simplon. Gipfelregion”, Gneiss, 2450 m, leg. H. Handel-Mazzetti, 29.7.1906 (WU 044259; http://herbarium.univie.ac.at/database/detail.php?ID=157653).

-Vitaliana primuliflora subsp. canescens O. Schwarz in Feddes Repert. 67: 24, 1963. nom. inval. (without type indication: ICBN 40.1), p. p. (plants from “Penninische und Lepontinische Alpen” only)

Distribution. Alps from the Great St. Bernard Pass to the Simplon Pass (Swiss canton of Valais, Italian provinces of Verbano and Valle d’Aosta).

A taxon restricted to the Pennine Alps has previously been recognized only by Chiarugi (1930) as Vitaliana primuliflora var. alpina f. orientalis subf. lepontina. Schwarz (1963) described two subspecies within his *Vitaliana primuliflora*, one of which (subsp. canescens) explicitly includes Chiarugi’s taxon, but also includes some plants from the Pyrenees. Schwarz (1963), however, failed to indicate a holotype, rendering the name (and thus also the later combination made by Vigo i Bonada 1983) invalid. The possibility of Schwarz having intended merely to provide a new name for Chiarugi’s taxon is ruled out by Schwarz’ explicit statement of “ssp. nov.” and is also made less likely by the additional Pyrenean area. To find a suitable name for a new combination, we must therefore return to Chiarugi’s subf. lepontina, which covers the same area as the present taxon, for which several syntypes are listed, and whose epithet *lepontina* is considerably more apt and less ambiguous than Schwarz’ “*canescens*”. We have chosen one of the syntypes which has detailed location information, which comes from a representative location, and whereon all the parts that are used for diagnosis within the species are preserved. The new lectotype is from the summit region of the Seehorn, south-east of the Simplon Pass in the Swiss canton of Valais.

Androsace vitaliana subsp. praetutiana (Buser ex Sündermann) Kress in Hegi, Ill. Fl. Mitt.-Eur. 5 (3): 2248d, 1967 ≡ Gregoria vitaliana var. praetutiana Buser ex Sündermann in Allg. Bot. Z. 22: 59. 1916 [basionym] ≡ Gregoria vitaliana subsp. praetutiana (Buser ex Sündermann) Bornm. in Mitt. Thür. Bot. Ver. 37: 6. 1927 ≡ Gregoria vitaliana f. praetutiana (Buser ex Sündermann) Lüdi in Hegi, Ill. Fl. Mitt.-Eur. 5 (3): 1789. 1927 ≡ Vitaliana primuliflora var. praetutiana (Buser ex Sündermann) Chiarugi in Nuov. Giorn. Bot. Ital. 37: 337, 1930 ≡ Vitaliana primuliflora subsp. praetutiana (Buser ex Sündermann) I. K. Ferguson in Taxon 18: 303, 1969 — Neotype (here designated): Gran Sasso d’Italia. Traversa di Portella, nei pascoli, leg. U. Martinelli 19.8.1893 (FI)

= *Vitaliana obtusifolia* O. Schwarz in Feddes Repert. 67: 26, 1963 — Type: Majella, Mte. Amaro, leg. H. Handel-Mazzetti (W).

Distribution. Mountains of Abruzzo: Corno Grande, Maiella and Sirente-Velino.

Sündermann (1916), in his description, ascribed subsp. praetutiana to Buser, although we are unaware of any publication by Buser dealing with *Androsace vitaliana*. Franz Sündermann was a commercial horticulturist in Lindau (Bavaria, Germany), and is unlikely to have maintained a herbarium himself. Buser’s herbarium is held at the Conservatoire et Jardin botaniques de la Ville de Genève (G) and does not include any specimens of *Androsace vitaliana* (Laurent Gautier, pers. comm.), suggesting that none of the material seen by the author of the protologue survives. For the purposes of typification, the location information is rather vague, being given simply as “in den Abruzzen” (i.e. Abruzzo, Italy), which covers the entire range of the subspecies. Since Chiarugi (1930) was the first to provide information on this taxon beyond the bald description of Sündermann, any neotype should be chosen from among the specimens seen by Chiarugi and identified by him as var. praetutiana. We have chosen a specimen that has detailed location information, which comes from a representative location, and whereon all the parts that are used for diagnosis within the species are preserved. Schwarz (1963) created a new epithet (*obtusifolia*) and selected a type specimen which Chiarugi did not investigate.

Androsace vitaliana subsp. sesleri (Buser ex Sündermann) Kress in Hegi, Ill. Fl. Mitt.-Eur. 5 (3): 2248d, 1967 ≡ Gregoria vitaliana var. sesleri Buser ex Sündermann in Allg. Bot. Z. 22: 59. 1916 [basionym] ≡ Gregoria vitaliana subsp. sesleri (Buser ex Sündermann) Bornm. in Mitt. Thür. Bot. Ver. 37: 6. 1927 ≡ Gregoria vitaliana f. sesleri (Buser ex Sündermann) Lüdi in Hegi, Ill. Fl. Mitt.-Eur. 5 (3): 1789. 1927 ≡ Vitaliana primuliflora subsp. sesleri (Buser ex Sündermann) Pignatti in Giorn. Bot. Ital. 111(1–2): 48. 1977 (1977) — Neotype (here designated): Italy, Trentino-Alto Adige: Trento, northern surroundings of Passo di San Pellegrino: little south of Pas de le Sele; 2485 m, 46°23'48’’ N, 11°45'50’’ E, leg. R. Flatscher & P. Schönswetter, 5.9.2010; cultivated in the Botanical Garden of the University of Innsbruck and prepared as herbarium sheet in spring 2011 (WU).

= Vitaliana primuliflora var. alpina f. orientalis subf. tridentina Chiarugi in Nuov. Giorn. Bot. Ital. 37: 337, 1930

-Vitaliana primuliflora subsp. primuliflora sensu I. K. Ferguson in Taxon 18: 302, 1969.

Distribution. South-eastern Alps from the Bergamo Alps (Italy) to the Carnic Alps (Italy and a small area in Austria), chiefly the Italian Dolomites.

As was the case for subsp. praetutiana, Sündermann (1916) ascribed subsp. sesleri to Buser and no type material has been identifiable. The location information for this taxon is stated as “die südtiroler und von da weiter östlich vorkommende Form” (“the form occurring in South Tyrol and further to the east”). Since no original material is available, a neotype is necessary, ideally from material collected in the source region of the material used for Sesler’s drawing, which has previously been used for typification of the species (see above). Sesler reports the location as “dans la montagne de S. Pellegrino” (cited in Ferguson 1969), which is a pass between Falcade and Moeno in the southern Dolomites. In the course of a dedicated search, the taxon has been collected in the northern surroundings of the Passo di San Pellegrino, and material from this population is chosen for the neotype.

Androsace vitaliana subsp. assoana (Laínz) Kress in Phyton (Horn) 13: 221. 1969 ≡ Vitaliana primuliflora subsp. assoana Laínz in Bot. Inst. Edt. Astur. Supplem. Cienc. 10: 199, 1964 ≡ *Androsace centriberica* (Kress) Kress in Primulaceen-Studien 15: 2. 1999 — Type: Spain, Teruel, “Sierra de Jabalambre” [*sic*], “in solo petroso cacuminis”, 2020 m, leg. J. Borja 3.8.1960, in herb. Borja (holotype: MAF; isotype: JE).

Distribution. Mountains of the Iberian Peninsula, excluding the Pyrenees.

Androsace vitaliana subsp. assoana var. assoana (Laínz) Kress in Primulaceen-Studien 13: 10. 1997 ≡ *Vitaliana intermedia* O. Schwarz in Feddes Repert. 67: 40. 1963 ≡ Vitaliana primuliflora subsp. assoana Laínz in Bot. Inst. Edt. Astur. Supplem. Cienc. 10: 199. 1964. ≡ Androsace centriberica subsp. assoana (Laínz) Kress in Primulaceen-Studien 15: 2. 1999 — Type: Spain, Teruel, “Sierra de Jabalambre” [*sic*], “in solo petroso cacuminis”, 2020 m, leg. J. Borja 3.8.1960 (holotype: MAF; isotype: JE).

Distribution. Sierra de Javalambre.

Androsace vitaliana subsp. assoana var. flosjugorum (Kress) Dixon, Guterm. & Schneew. stat. nov. ≡ Androsace vitaliana subsp. flosjugorum Kress in Primulaceen-Studien 13: 9. 1997 — Type: Spain, Cantabria, Peña Prieta, “zwischen Alto de Cubil de Can und Alto del Tio Celestino”, 2200–2450 m, 8.6.1967. “In herb. Kress V41+”.

= Androsace centriberica subsp. maragatorum Kress in Primulaceen-Studien 15: 2. 1999 — Type: Spain, León, Montes de León, “vom Paß im OOSO von Peñalba de Santiago - (14 km sssö Ponferrada) auf der S-Seite - des Baches Santiago ca. 150 m abwärts - 1450–1300 m”, in herbario Kress.

Distribution. Cordillera Cantábrica and Montes de León.

Androsace vitaliana subsp. assoana var. centriberica Kress in Primulaceen-Studien 13: 10. 1997 ≡ Androsace vitaliana subsp. aurelii Luceño in Anales Jard. Bot. Madrid 56: 165. 1998 — Type: Spain, Ávila, Sierra de El Barco, cuerda de la Covacha del Losat, 30T TK 8056, 2300 m, en pastizales psicroxerófilos, 27.7.1983. Leg. M. Luceño, 260028 (MA).

≡ Androsace centriberica subsp. centriberica [per Kress in Primulaceen-Studien 15: 2. 1999]

Distribution. Sistema Central (Sierra de Gredos and probably also Sierra de Guadarrama).

This variety is found in the Sierra de Gredos and we assume on geographical grounds that this is also the taxon from the Sierra de Guadarrama, from where we have not seen any material.

Androsace vitaliana subsp. assoana var. nevadensis (Chiarugi) Kress in Primulaceen-Studien 13: 10. 1997 ≡ Vitaliana primuliflora var. nevadensis Chiarugi in Nuov. Giorn. Bot. Ital. 37: 338, 1930 [basionym] ≡ Androsace vitaliana subsp. nevadensis (Chiarugi) Luceño in Anales Jard. Bot. Madrid 56: 165. 1998 (1998) — Lectotype (here designated): Spain, Sierra Nevada, “région niveuse, au *Picacho de Veleta*”, 5.7.1851. J. Gay in E. Bourgeau, *Plantes d’Espagne*. Herbarium Webbianum, 120348 (FI).

= *Vitaliana congesta* O. Schwarz in Feddes Repert. 67: 25, 1963 = *Androsace congesta* (O. Schwarz) Kress in Primulaceen-Studien 15: 2. 1999 — Type: Spain, Sierra Nevada, Cerro de Medio Dia, 2800 m, leg. Dürck (M).

Distribution. Sierra Nevada.

The basionym of this name, Chiarugi’s (1930) var. nevadensis, has no explicitly designated type specimen; Chiarugi lists six specimens which he consulted, and the lectotype must be chosen from among these six syntypes. We have been able to see detailed photographs of five of the six specimens, and only one of those bears a significant number of flowers. We have therefore chosen this specimen to be the lectotype. Schwarz (1963) used the epithet *congesta* and chose a specimen not investigated by Chiarugi to be the type.
